# Detection of the DNA methylation of seven genes contribute to the early diagnosis of lung cancer

**DOI:** 10.1007/s00432-023-05588-z

**Published:** 2024-02-05

**Authors:** Chaoxiang Du, Lijie Tan, Xiao Xiao, Beibei Xin, Hui Xiong, Yuying Zhang, Zhonghe Ke, Jun Yin

**Affiliations:** 1 Department of Thoracic Surgery, Cancer Center, Zhongshan Hospital of Fudan University, Shanghai, China; 2 Zhongshan Hospital (Xiamen), Fudan University, Xiamen, China; 3 School of Physics, Changchun University of Science and Technology, Changchun, 130022 China; 4 Shanghai Rightongene Biotechnology Co. Ltd., Shanghai, 201403 China

**Keywords:** Lung cancer, DNA methylation, Diagnosis, Stage, Ground-glass nodules

## Abstract

**Background:**

Low-dose Computed Tomography (CT) is used for the detection of pulmonary nodules, but the ambiguous risk evaluation causes overdiagnosis. Here, we explored the significance of the DNA methylation of 7 genes including *TAC1*, *CDO1*, *HOXA9*, *ZFP42*, *SOX17*, *RASSF1A* and *SHOX2* in the blood cfDNA samples in distinguishing lung cancer from benign nodules and healthy individuals.

**Method:**

A total of 149 lung cancer patients [72 mass and 77 ground-glass nodules (GGNs)], 5 benign and 48 healthy individuals were tested and analyzed in this study. The lasso-logistic regression model was built for distinguishing cancer and control/healthy individuals or IA lung cancer and non-IA lung cancer cases.

**Results:**

The positive rates of methylation of 7 genes were higher in the cancer group as compared with the healthy group. We constructed a model using age, sex and the ΔCt value of 7 gene methylation to distinguish lung cancer from benign and healthy individuals. The sensitivity, specificity and AUC (area under the curve) were 86.7%, 81.4% and 0.891, respectively. Also, we assessed the significance of 7 gene methylation together with patients’ age and sex in distinguishing of GGNs type from the mass type. The sensitivity, specificity and AUC were 77.1%, 65.8% and 0.753, respectively. Furthermore, the methylation positive rates of *CDO1* and *SHOX2* were different between I-IV stages of lung cancer. Specifically, the positive rate of *CDO1* methylation was higher in the non-IA group as compared with the IA group.

**Conclusion:**

Collectively, this study reveals that the methylation of 7 genes has a big significance in the diagnosis of lung cancer with high sensitivity and specificity. Also, the 7 genes present with certain significance in distinguishing the GGN type lung cancer, as well as different stages.

**Supplementary Information:**

The online version contains supplementary material available at 10.1007/s00432-023-05588-z.

## Introduction

Lung cancer is the leading cause of cancer-related mortality globally, with about 2.2 million incidences and 1.8 million deaths in 2020 (Hughes et al. 2022). Late diagnosis is largely responsible for its extremely high mortality rate (Ji et al. 2023). The 5-year survival rate for patients with stage I disease is about 81%–85% while it decreases in 15%–19% for patients with higher stages (Begum et al. 2011; Blandin Knight et al. 2017). Therefore, early diagnosis of lung cancer is important, which can help improve the outcome of patients.

Low-dose Computed Tomography (CT) is widely used for detection of pulmonary nodules, but the ambiguous risk evaluation often causes overdiagnosis and radioactivity. To this end, researchers have made efforts all the time in seeking blood markers for early diagnosis of lung cancer, with the most intensively investigated biomarkers including squamous cell carcinoma antigen (SCC-Ag), cytokeratin 19 fragment (CYFRA 21–1), carcinoembryonic antigen (CEA), and neuron-specific enolase (NSE) (Hu et al. 2023). Regretfully, the low sensitivity decreases the performances of those biomarkers. With the increased research focusing on the field of epigenetics, which regulates gene expression without altering the DNA sequence, its crucial roles in the diagnosis of lung cancer have been largely uncovered. DNA methylation is a well-known epigenetic alteration that involves the covalent addition of a methyl group to the cytosine residue of CpG dinucleotides, leading to transcriptional repression (Ansari et al. 2016). In lung cancer, several studies have published their data to support the potentially high values of gene methylation in the early diagnosis of lung cancer using the circulating free DNA (cfDNA) samples. For instance, Hu et al. (2023) developed a “7-DMR model” (7 differentially methylated genes (*HOXB4*, *HOXA7*, *HOXD8*, *ITGA4*, *ZNF808*, *PTGER4*, and *B3GNTL1*) to distinguish lung cancers from benign nodules, achieving the sensitivities of 89%/92%, specificities of 94%/100%, and accuracies of 90%/94% in the discovery cohort and validation cohort. Chen et al. (2020) demonstrated that the combination of *CDO1*, *SOX17*, and *HOXA7* had the ability in distinguishing the smallest lung nodules among 1.1–2.0 cm (sensitivity 74%; specificity, 93%), while the combination of *CDO1*, *TAC1*, and *SOX17* was best in tumor sizes < 1.0 cm (sensitivity 71%; specificity, 82%). However, the performance of these models needs to be improved, leaving the combination of gene methylation panel as a problem demanding prompt solution.

Following a large literature review (Wrangle et al. 2014; Yin et al. 2012; Yang et al. 2019; Li et al. 2020; Chen et al. 2020; Hulbert et al. 2017; Brait et al. 2012; Di Vinci et al. 2012; Hwang et al. 2015; Ooki et al. 2017; Zeng et al. 2019; Zhao et al. 2016; Song et al. 2015), we explored the performance of the DNA methylation of 7 genes including *TAC1*, *CDO1*, *HOXA9*, *ZFP42*, *SOX17*, *RASSF1A* and *SHOX2* in the blood cfDNA samples in distinguishing lung cancer from benign nodules and healthy individuals.

## Materials and methods

### Sample collection

From July 2022 to December 2022, 237 blood samples collected from 237 individuals were included in this study, including 92 patients with mass diseases, 92 patients with ground-glass nodules (GGNs) and 53 healthy individuals. Inclusion criteria: aged > 18 years old; individuals with pulmonary nodule (for mass and GGN individuals); signed the informed consent; Exclusion criteria: combined with other tumors. Lung cancer patients who received any pretreatment therapy, including chemotherapy or radiotherapy, or had a history of other malignancies were not included. All patients received curative-intent resection. The blood sample was obtained from each patient prior to surgery and was immediately processed to isolate plasma. All patients with pathologically confirmed malignant lesions were staged according to the revised TNM guidelines classification criteria (Detterbeck et al. 2017). Patients with lung cancer were included as cancer group, those with histologically benign lesions as the control group. Plasma samples of 49 healthy volunteers were also considered as the control group.

### DNA isolation and quantitative multiplex methylation-specific PCR (qMSP)

3 mL plasma was collected from each individual and the cell-free nucleic acid was extracted using the plasma-free DNA extraction kit (Shanghai Rightongene Biotechnology Co. Ltd., Shanghai, China) based on the manufacturer’s directions. Then, the DNA was eluted by 60 μL eluent buffer, which was used as a template for subsequent experiments. DNA concentration and purity were evaluated using a Qubit 3.0 Fluorometer (Thermo Fisher Scientific, Waltham, MA, USA). DNA was bisulfite-converted using the DNA Methylation kit (Shanghai Yuanqi, Shanghai, China). For methylation analysis, EpiTect MethyLight Master Mix (Qiagen) was used, together with fluorescent dye-(Chen et al. 2020) labeled probes, 50 ng of bisulfite-converted DNA and 100–300 nM of each primer. The DNA methylation of 7 genes, including *TAC1*, *CDO1*, *HOXA9*, *ZFP42*, *SOX17*, *RASSF1A* and *SHOX2*) in three multiplex qMSP assays were detected, with *β-actin* (*ACTB*) as the reference gene. Δ*C*_t _ was calculated as follows: Δ*C*_t _= the Ct value (target gene)—the *C*_t _ value (reference gene). The mixture DNA sample extracted from NCI-H596 and NCI-H460 at a ratio of 1:1 was used as a positive control. Buffy-coat gDNA extracted from the blood samples of healthy individuals and verified by the Sanger sequencing was used as the negative control. The primers of DNA methylation were synthesized according to an applied patent (No. 2022114063829) and the number of CpGs covered was listed in Supplementary Table 1. The sample was considered as successfully detected when the *C*_t _ value of the reference gene (*ACTB*) was < 35. Based on this, the gene was defined as methylated when the *C*_t _ value < 42. The Ct value was defined as 45 for the negative methylated gene in samples as the cycle of the PCR assay was set as 45.

### Construction of models for lung cancer diagnosis and IA stage prediction

A lasso-logistic regression model was built for distinguishing cancer and control/healthy individuals or IA lung cancer and non-IA lung cancer cases. The model was visualized by receiver operating characteristic (ROC) curves, assessed through the area under the curve (AUC). Logistic regression analysis was performed in R open-source software version 4.0.2 and the pROC package was implemented for ROC analysis. Consideration of the variables including age, sex, and the ΔCt or the status of 7 gene methylation, we construct a model to distinguish lung cancer patients from the benign and healthy individuals with the best performance, with 5 benign and 48 healthy individuals as the control group. The formula was as follows: *pre* = – 0.055613age + 0.044842ΔCt (TAC1FAM) + 0.033004ΔCt (HOXA9) + 0.055091ΔCt(ZFP42) + 0.014456ΔCt(RASSF1A)-0.021013ΔCt(SHOX2). For the IA stage prediction model, the formula was as follows: *pre* = 0.02510age-0.57283 sex(male = 1) + 0.37636CDO1(positive = 1) + 0.376358ZFP42(positive = 1) + 0.17867SOX17(positive = 1) – 0.16980RASSF1A(positive = 1) + 0.59558SHOX2(positive = 1).

### Statistical analysis

All other statistical analyses were performed in IBM SPSS Statistics software for Windows version 24.0 (IBM Corporation, Armonk, NY, USA). Reported *P* values were 2-sided. *P* < 0.05 was considered to be significantly different. * represents *P* < 0.05, ** represents *P* < 0.01, and *** represents *P* < 0.001.

## Results

### Patient characteristics

**Fig. 1:**
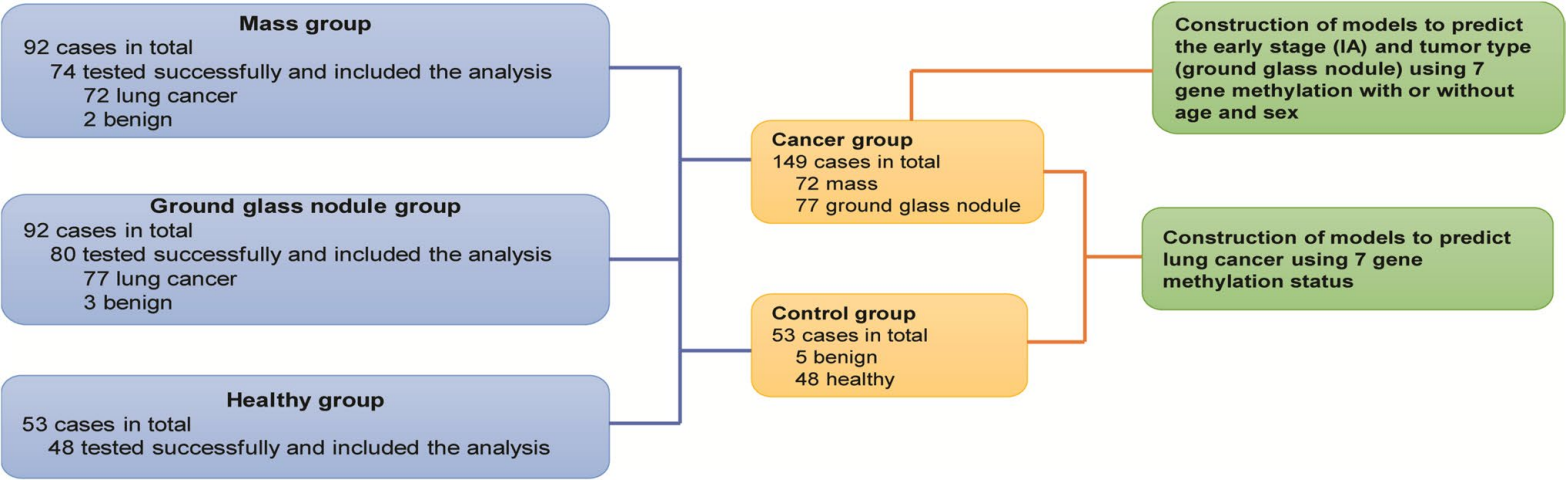
Flowchart for finding lung cancer candidate diagnostic biomarkers

A total of 237 blood samples collected from 237 individuals were included in this study, including 92 patients with mass diseases, 92 patients with GGNs and 53 healthy individuals, among which 202 samples were tested successfully and included in the next analysis. Detailly, 74 cases of the mass group were tested successfully, including 72 patients with lung cancer and 2 patients with benign nodules, 80 cases of the GGNs group were tested successfully, including 77 patients with lung cancer and 3 patients with benign nodules, and 48 cases of the healthy group were tested successfully (Fig. 1).

In total, 149 lung cancer were included, including 72 patients from the mass group and 77 patients from the GGNs group. As shown in Table 1, 51 (70.8%) and 21 (29.2%) male cases were found in the lung cancer patients from the mass and GGNs groups, respectively. Compared with the mass group, more female patients were in the GGNs group (53.2% vs. 29.2%, *P* = 0.006), together with a lower average age (60.7 ± 10.6 vs. 67.5 ± 8.8, *P* < 0.0001). In addition, more squamous carcinoma cases were found in the lung cancer patients from the mass group, together with less adenocarcinoma cases according to the histopathology (*P* < 0.0001). Moreover, 24 cases (35.8%) were diagnosed with IA stage for lung cancer patients from the mass group, while it increased to 47 (70.1%) for lung cancer patients from the GGNs group (*P* < 0.0001). Taken together, the clinical features such as sex, age, histopathology and pTNM stage were significantly different between the lung cancer patients from the mass and GGNs groups.

**Table 1:** The clinical information of the 149 lung cancer patients

Clinicopathological features	Lung cancer (*n* = 149)	Mass group (*n* = 72)	GGNs group (*n* = 77)	*P*
Sex (*n*, %)				
Male	87 (58.4)	51 (70.8)	36 (46.8)	0.006
Female	62 (41.6)	21 (29.2)	41 (53.2)	
Age (mean ± SD)	64.0 ± 10.3	67.5 ± 8.8	60.7 ± 10.6	< 0.0001
Histopathology (*n*, %)				
Large cell cancer	1 (0.7)	1 (1.4)	0 (0.0)	
Squamous carcinoma	27 (18.2)	19 (26.8)	8 (10.4)	
Neuroendocrine neoplasm	3 (2.0)	3 (4.2)	0 (0.0)	
Adenocarcinoma	108 (73.0)	41 (57.7)	67 (87.0)	0.004
Adenosquamous carcinoma	5 (3.4)	4 (5.6)	1 (1.3)	
Adenocarcinoma	108 (73.0)	41 (57.7)	67 (87.0)	
pTNM stage (*n*, %)				
IA	71 (53.0)	24 (35.8)	47 (70.1)	< 0.0001
Non-IA	63 (47.0)	43 (64.2)	20 (29.9)	

### The value of 7 gene methylation in the diagnosis of lung cancer

**Fig. 2:**
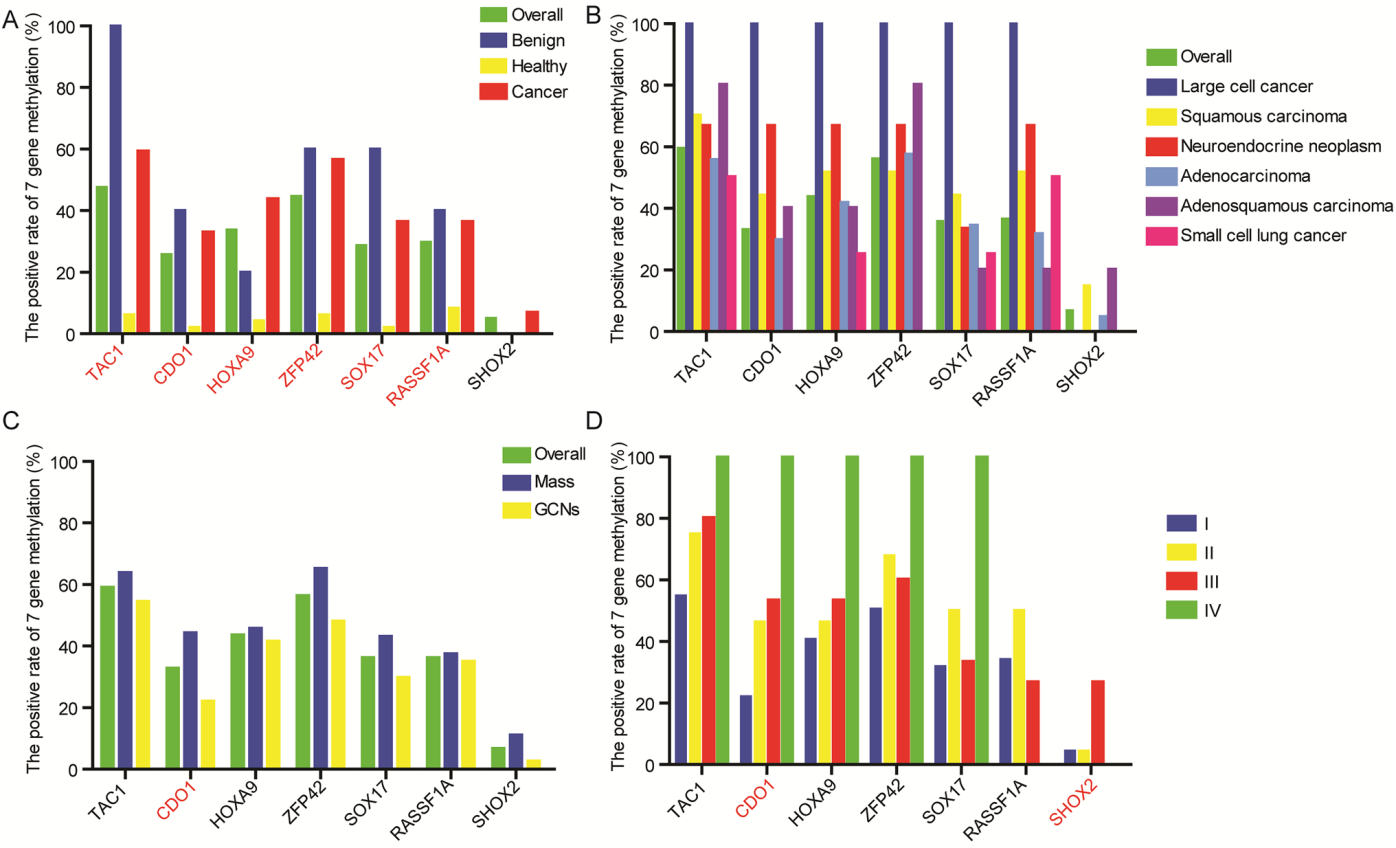
The positive rate of 7 gene methylation in different groups. **A** Benign, healthy and cancer patients. **B** Lung cancer patients with different histopathology. **C** Lung cancer of mass and GGN types. **D** Lung cancer patients with different stages. Gene marked red refers to the positive rate of this gene methylation shows a significant difference between groups (*P* < 0.05)

Then, we explored the diagnosis value of the DNA methylation status of 7 genes including *TAC1*, *CDO1*, *HOXA9*, *ZFP42*, *SOX17*, *RASSF1A* and *SHOX2* in lung cancer. The positive rates of the methylation of all 7 genes were significantly higher in the cancer group as compared with the healthy group (Fig. 2A). All 5 cases (100%) with benign nodule were positive for *TAC1* methylation, while 2 (40.0%), 1 (20.0%), 3 (60.0%), 3 (60.0%), 2 (40.0%) and 0 (0%) of the 5 cases were positive for the methylation of *CDO1*, *HOXA9*, *ZFP42*, *SOX17*, *RASSF1A* and *SHOX2*, respectively (Fig. 2A). In addition, no obvious difference in the DNA methylation status of 7 genes was observed in lung cancer patients with different histopathology, as shown in Fig. 2B.

**Fig. 3:**
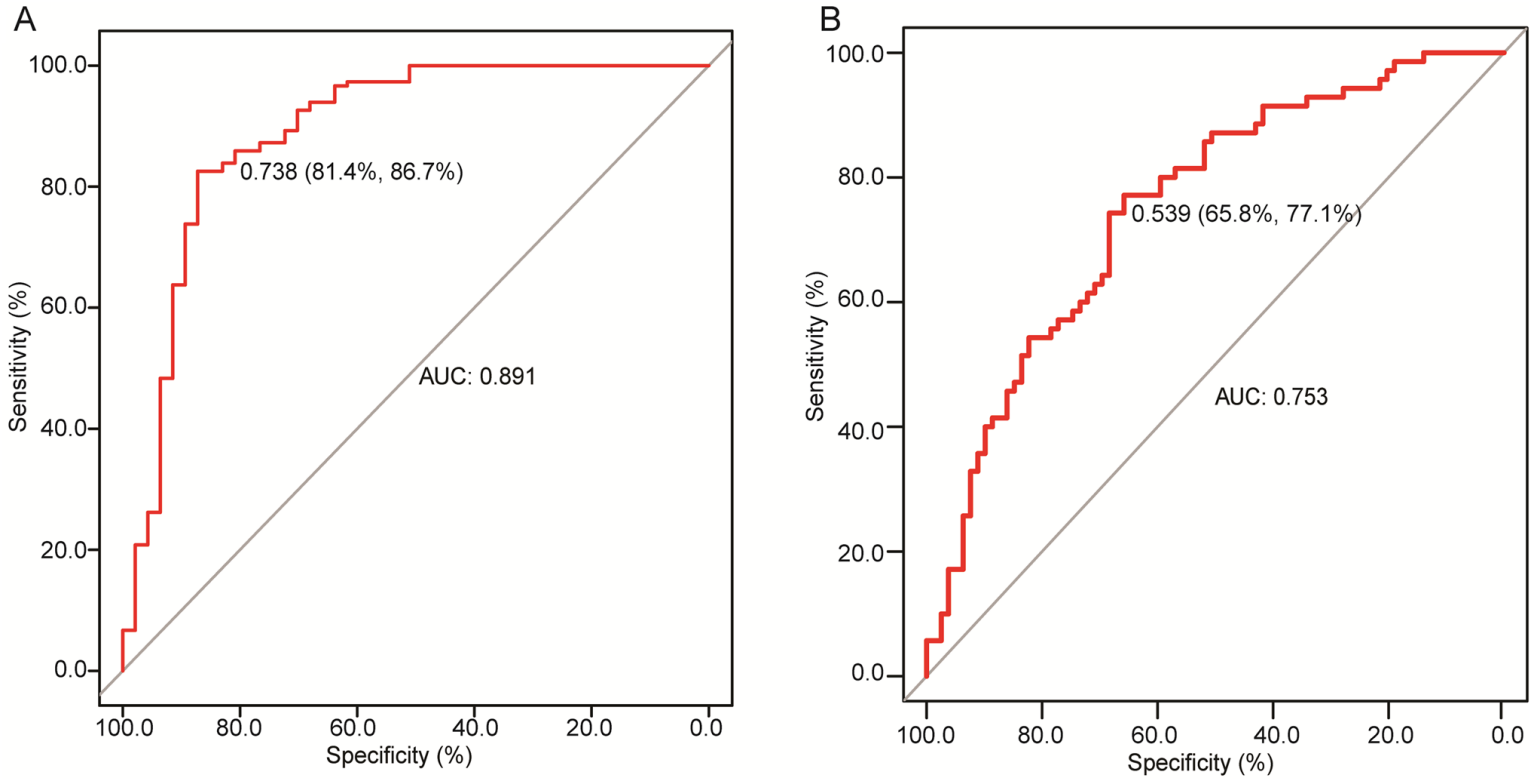
Evaluation of the accuracy of the diagnostic model of the combination of the DNA methylation of seven genes in lung cancer. ROC curves showed the sensitivity, specificity and AUC of these 7 gene methylation statuses in distinguishing **A** lung cancer from benign and healthy individuals, and **B** GGNs type lung cancer from mass type

Subsequently, we assessed the performance of a single gene in the diagnosis of lung cancer. ROC curves showed the AUC of a single gene was not good (0.546–0.716), as shown in supplementary Fig. 1A. Thus, we construct a model to distinguish lung cancer patients from benign and healthy individuals using the status of 7 gene methylation. The 5 benign and 48 healthy individuals were considered as the control group. Using the logistic regression, the model was constructed using the ΔCt values of the 7 genes together with patient’s age and sex (male = 0, female = 1), achieving a sensitivity, specificity, and AUC of 86.7%, 81.4% and 0.891, respectively (Fig. 3A). These results revealed a potential role of 7 gene methylation in the diagnosis of lung cancer.

### The value of 7 gene methylation in distinguishing the GGNs type of lung cancer from mass type

Moreover, we compared the DNA methylation status of 7 genes (*TAC1*, *CDO1*, *HOXA9*, *ZFP42*, *SOX17*, *RASSF1A* and *SHOX*2) in lung cancer cases from the mass and GGNs groups. Compared with the GGNs-original lung cancer, patients with mass original showed higher positive rates in *CDO1* (*P* = 0.006) and *RASSF1A* methylation (*P* = 0.08), respectively (Fig. 2C). Also, we assessed the performance of a single gene in the diagnosis of lung cancer. ROC curves showed the AUC of a single gene was not good (0.421–0.789), as shown in supplementary Fig. 1B.

we construct a model to predict whether the lung cancer patients from mass or GGN. Using the logistic regression, the model was constructed using the methylation status of 7 genes together with the patient’s age and sex (male = 0, female = 1) with a sensitivity, specificity, and AUC of 77.1%, 65.8% and 0.753, respectively (Fig. 3B). These results revealed a potential role of the methylation of the 7 genes in distinguishing GGNs type lung cancer from mass type.

### The value of 7 gene methylation in the diagnosis of the early stage of lung cancer

**Fig. 4:**
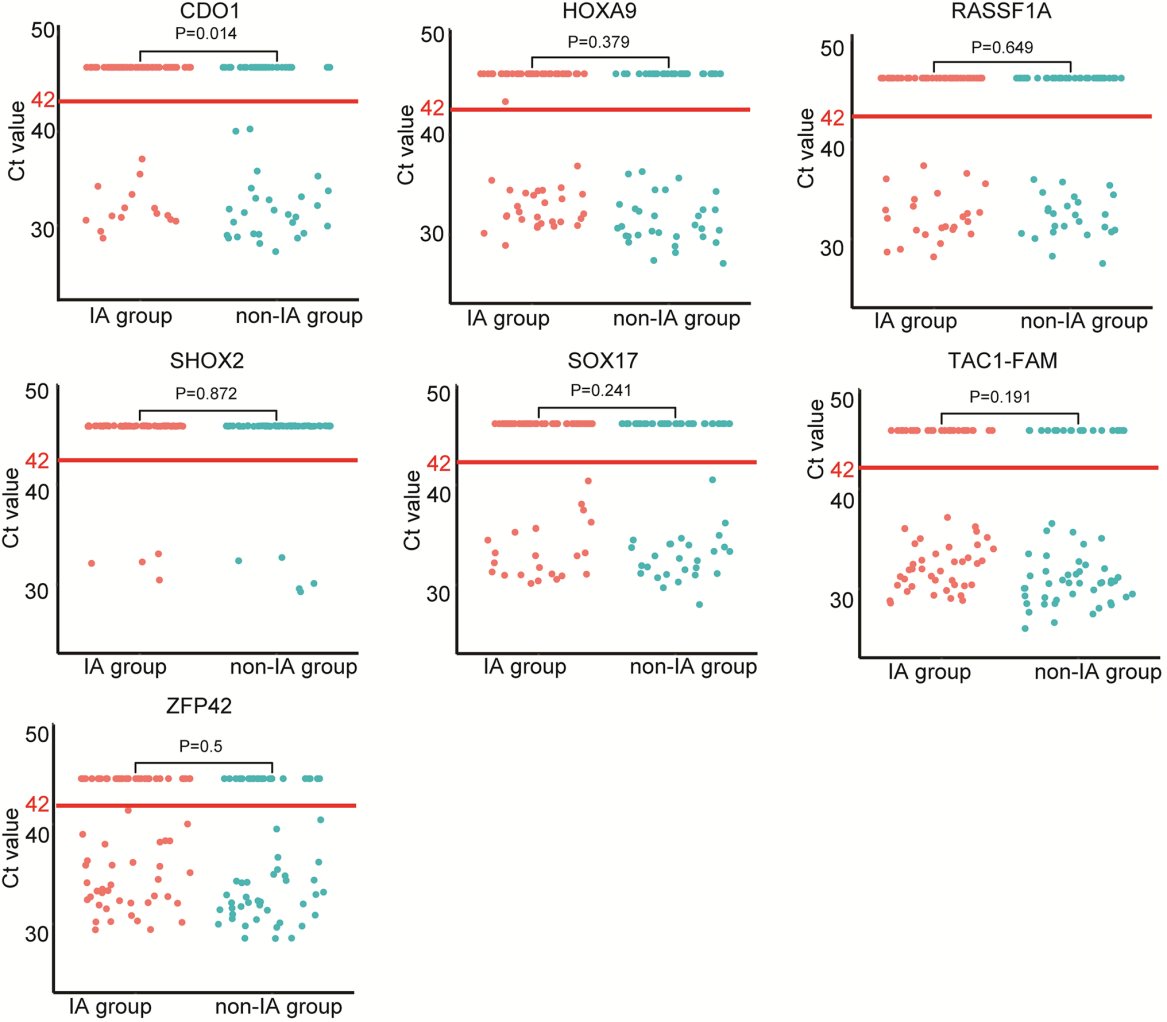
The DNA methylation status of seven genes in different groups. The positive rate of CDO1 methylation was significantly higher in the non-IA group as compared with the IA group (42.2% vs. 21.1%, *P* = 0.014)

Prediction of the tumor size concerns the resection range, thus it is essential to accurately predict the tumor size before surgery. Here, we compared the DNA methylation status of 7 genes including *TAC1*, *CDO1*, *HOXA9*, *ZFP42*, *SOX17*, *RASSF1A* and *SHOX2* in lung cancer cases with I–IV stages. A total of 91, 28, 15 and 1 patients with I, II, III and IV stages of lung cancer were included in this analysis, respectively. The results showed that the methylation rate of *CDO1* and *SHOX2* showed significantly different between the I-IV stages of lung cancer (Fig. 2D). In addition, we compared the methylation status of these 7 genes in patients with IA and non-IA stage. The positive rate of *CDO1* methylation was significantly higher in the non-IA group as compared with the IA group (42.2% vs. 21.1%, *P* = 0.014), while the methylation status of other 6 genes showed no significant difference (Fig. 4). This result suggested that gene methylation may contribute to find lung cancer patients with different stages, which may show a guiding value in the resection range of lung cancer.

## Discussion

In this study, we explored the significance of the DNA methylation of 7 genes including *TAC1*, *CDO1*, *HOXA9*, *ZFP42*, *SOX17*, *RASSF1A* and *SHOX2* in the blood cfDNA samples in distinguishing lung cancer from benign nodules and healthy individuals. Our results first reveal that the methylation of these 7 genes has a big significance in the diagnosis of lung cancer and achieved a diagnostic model with high sensitivity and specificity.

With the improvement in CT scanners and the increasing awareness of physical examination, more pulmonary nodules are identified in 1.6 million patients per year in the US (Mazzone and Lam 2022). At least 95% of all pulmonary nodules identified are benign, most often granulomas or intrapulmonary lymph nodes (Sun et al. 2020). This together with the radiation caused by the CT drive the development of ideal biomarkers which are expected to be further found in biological fluids for the non-invasive diagnosis of cancers, including lung cancer (Li et al. 2022b). Some lung cancer-related markers, including CEA, carbohydrate antigen 125 (CA125), cytokeratin 19 fragment (CY211), NSE, and SCC, have been widely reported. Among these biomarkers, the combination of CEA, CA125, CY211 and SCC showed the best performance with a sensitivity of 83.3%, a specificity of 62.9% and an AUC of 0.867 (Yang et al. 2018). In addition, Muller et al. (Muller et al. 2017) constructed a model that includes the variables related to smoking history and nicotine addiction, medical history, family history of lung cancer, and lung function (forced expiratory volume in 1 s [FEV1]) with excellent discrimination (concordance (*c*)-statistic = 0.85). Ajona et al. (Ajona et al. 2021) developed a diagnostic model based on the quantification in plasma of complement-derived fragment C4c, CYFRA 21–1 and C-reactive protein (CRP) with an AUC of 0.86 and a specificity of 92%. Among the multiple biomarkers, DNA methylation shows good performance (P. Li et al. 2022a; Magenheim et al. 2022; Liang et al. 2021). *SOX17*, *TAC1*, *CDO1*, *HOXA9* and *ZFP4*2 were the 5 genes that were identified in the Cancer Genome Atlas (TCGA) with highly prevalent DNA methylation in lung squamous and adenocarcinoma, but not in normal lung tissue (Cancer Genome Atlas Research, 2012; Wrangle et al. 2014; Diaz-Lagares et al. 2016). Hulbert et al. (Hulbert et al. 2017) reported that the combination of *CDO1*, *TAC1* and *SOX17* in plasma showed a sensitivity, specificity and AUC of 86%, 78% and 77% in the diagnosis of non-small cell lung cancer with stage I and IIA from the individuals with non-cancer. Abou-Zeid et al. (Abou-Zeid et al. 2023) reported that the methylation level of *HOXA9* was significantly higher in NSCLC patients than controls (*P* > 0.001). Liu et al. (Liu et al. 2017) used the Mate-analysis through the systematic literature search yielded a total of 33 studies including a total of 4801 subjects (2238 patients with lung cancer and 2563 controls) and covering 32 genes. Their findings demonstrated that *SOX17* (sensitivity: 84%, specificity: 88%), *CDO1* (sensitivity: 78%, specificity: 67%), *ZFP42* (sensitivity: 87%, specificity: 63%) and *TAC1* (sensitivity: 86%, specificity: 75%) were the superior genes. In addition, Gao et al. (Gao et al. 2022) reported that the promoter methylation level of *SHOX2* and *RASSF1A* was significantly higher in tumor samples at stage I-II than that in normal samples. Thus, the 7 genes (*TAC1*, *CDO1*, *HOXA9*, *ZFP42*, *SOX17*, *RASSF1A* and *SHOX2*) were included in this study and considered as the study subjects. Recently, Hu et al. (Hu et al. 2023) constructed a noninvasive 7-DMR model (7 differentially methylated genes, *HOXB4*, *HOXA7*, *HOXD8*, *ITGA4*, *ZNF808*, *PTGER4*, and *B3GNTL1*) to discriminate lung cancers and non-lung cancers including benign lung diseases and healthy controls, with a sensitivity of 81% and a specificity of 98%. Herein, we explored the value of other 7 genes in the diagnosis of lung cancer and achieved an increased sensitivity (from 81% to 86.7%) as compared with the 7-DMR model. We focused on the model’s sensitivity to distinguish lung cancer and benign lung diseases and healthy controls, as this model aimed to find the potential cancer patients whom were recommended for further examination to confirm cancers.

Recently, the increased number of GGNs attracted unprecedented attention. GGNs can be further classified into pure GGN (pGGN) and part-solid nodule according to the presence of solid components. About 20% of lung adenocarcinomas manifested as pGGN and showed favorable prognosis as compared with solid lung cancer (Mazzone and Lam 2022; Chang et al. 2013; Heidinger et al. 2017). Thus, the identification of the solid or pGGN is of importance. Herein, we demonstrated the value of 7 genes together with patients’ age and sex in distinguishing GGN and solid lung cancer.

In addition, correct prediction of the size of pulmonary nodule is crucial for the following surgery, which directly concern the extent of surgical resection. To this end, we compared the DNA methylation status of 7 genes including *TAC1*, *CDO1*, *HOXA9*, *ZFP42*, *SOX17*, *RASSF1A* and *SHOX2* in lung cancer cases with different stages. The results showed that the methylation rate of *CDO1* and *SHOX2* showed significantly different between the I-IV stages of lung cancer. In addition, the positive rate of *CDO1* methylation was significantly higher in the non-IA group as compared with the IA group. These results indicated the *CDO1* and *SHOX2* methylation have a certain significance for tumor staging of lung cancer.

Collectively, this study reveals that the methylation of 7 genes (*TAC1*, *CDO1*, *HOXA9*, *ZFP42*, *SOX17*, *RASSF1A* and *SHOX2*) has a big significance in the diagnosis of lung cancer and achieved a diagnostic model with high sensitivity and specificity. Also, the 7 genes present with certain significance in distinguishing the GGN type lung cancer, as well as different stages. Further study with larger size samples will be carried out to further explore the significance of DNA methylation in distinguishing the various stages of lung cancer.

### Supplementary Information

Below is the link to the electronic supplementary material.

Supplementary Figure 1 Evaluation of the performance of single gene in (A) the diagnosis of lung cancer and (B) distinguishing patients from IA stage.

Supplementary file2 (DOCX 15 kb)

## Data Availability

The original contributions presented in the study can be directed to the corresponding author.
