# Supplementary figures and images for: Detection of the DNA methylation of seven genes contribute to the early diagnosis of lung cancer
Source: J Cancer Res Clin Oncol. 2024 Feb 5;150(2):77. doi: 10.1007/s00432-023-05588-z (PMC10844440; doi:10.1007/s00432-023-05588-z)

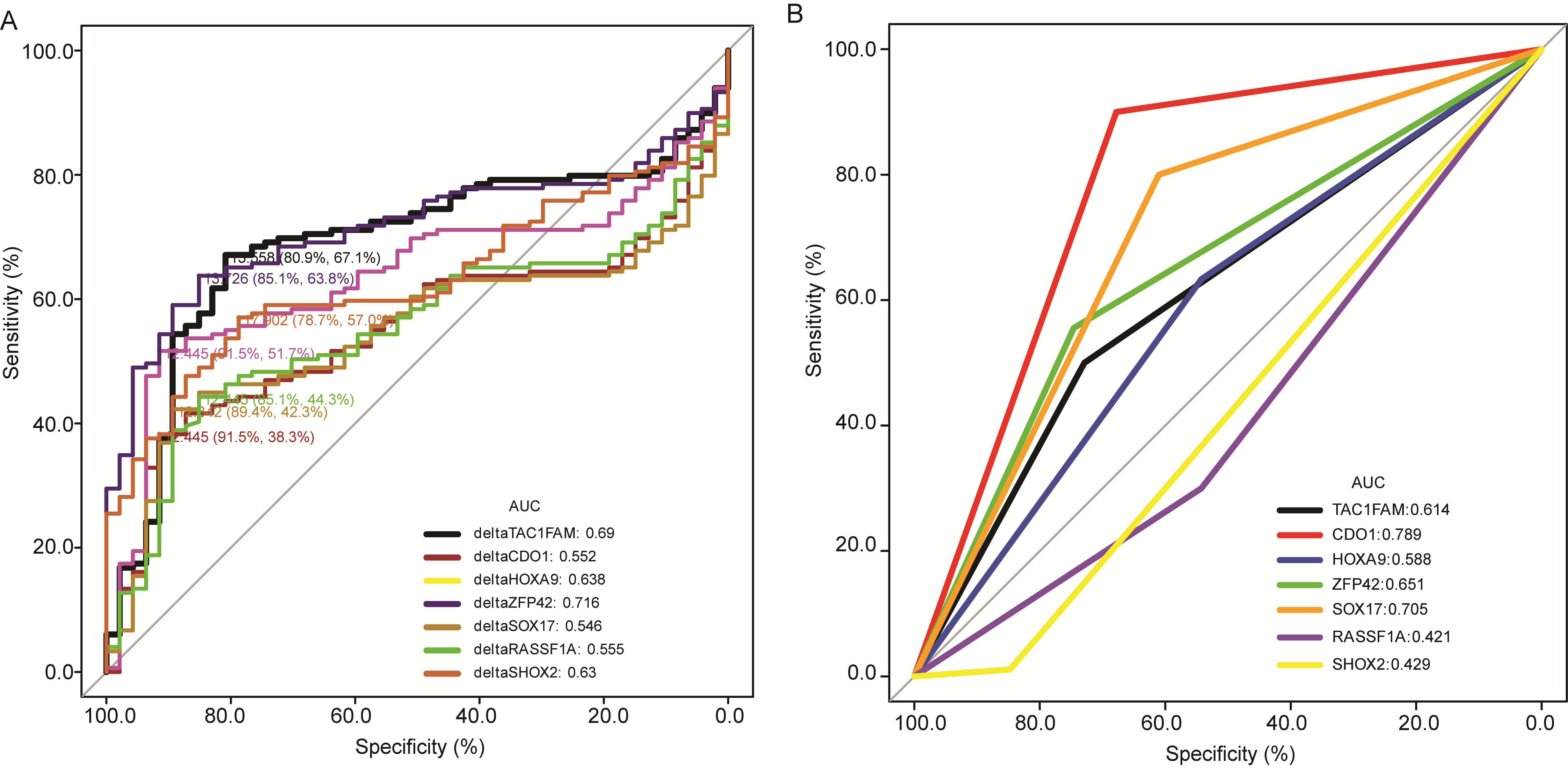

Supplement: Supplementary file 1 — Supplementary Figure 1 Evaluation of the performance of single gene in (A) the diagnosis of lung cancer and (B) distinguishing patients from IA stage. [file 432_2023_5588_MOESM1_ESM.tif]
