# Supplementary material for: Detection of the DNA methylation of seven genes contribute to the early diagnosis of lung cancer
Source: J Cancer Res Clin Oncol. 2024 Feb 5;150(2):77. doi: 10.1007/s00432-023-05588-z (PMC10844440; doi:10.1007/s00432-023-05588-z)
Supplement: Supplementary file 2 — Supplementary file2 (DOCX 15 kb) [file 432_2023_5588_MOESM2_ESM.docx]

**Supplementary Table 1** The number of CpGs covered by the primers and probes of each gene.

| Gene | The number of CpGs covered | | |
| --- | --- | --- | --- |
|  | Frimer number | Reverse number | Probe |
| *TAC1* | 4 | 4 | 4 |
| *CDO1* | 5 | 3 | 4 |
| *HOXA9* | 4 | 4 | 2 |
| *ZFP42* | 4 | 3 | 4 |
| *SOX17* | 4 | 5 | 4 |
| *RASSF1A* | 4 | 3 | 2 |
| *SHOX2* | 3 | 4 | 4 |
